# A Method for 3D Immunostaining and Optical Imaging of the Mouse Brain Demonstrated in Neural Progenitor Cells

**DOI:** 10.1371/journal.pone.0072039

**Published:** 2013-08-06

**Authors:** Jacqueline A. Gleave, Jason P. Lerch, R. Mark Henkelman, Brian J. Nieman

**Affiliations:** 1 Mouse Imaging Centre, Hospital for Sick Children, Toronto, Ontario, Canada; 2 Department of Biological Sciences, University of Toronto, Toronto, Ontario, Canada; 3 Department of Medical Biophysics, University of Toronto, Toronto, Ontario, Canada; The University of Chicago, United States of America

## Abstract

It is important to understand changes in cell distribution that occur as a part of disease progression. This is typically achieved using standard sectioning and immunostaining, however, many structures and cell distribution patterns are not readily appreciated in two-dimensions, including the distribution of neural stem and progenitor cells in the mouse forebrain. Three-dimensional immunostaining in the mouse brain has been hampered by poor penetration. For this reason, we have developed a method that allows for entire hemispheres of the mouse brain to be stained using commercially available antibodies. Brains stained for glial fibrillary acidic protein, doublecortin and nestin were imaged in three-dimensions using optical projection tomography and serial two-photon tomography. This staining method is simple, using a combination of heat, time and specimen preparation procedures readily available, so that it can be easily implemented without the need for specialized equipment, making it accessible to most laboratories.

## Introduction

The spatial distribution of various cell types or proteins is fundamental to understanding normal and pathological processes in the brain. Many studies use mouse models to probe the importance of certain cells or proteins and then rely on histological sectioning and antibody staining to generate representative two-dimensional (2D) sections. However, many structures or cell distributions, such as migrating neural progenitor cells, vasculature, and branching axonal connections, are not readily appreciated in 2D. While alignment of serially-stained sections is a possible workaround for this problem, it is difficult, laborious and impractical for routine use. Furthermore, comparison between control and experimental groups in a study routinely requires cutting and identification of equivalent sections in multiple specimens, a subjective process that can be difficult even in simple cases.

For these and other reasons, several optical imaging methods have been developed that enable imaging of the mouse brain directly in three-dimensions (3D) [1]–[4]. Examples include optical projection tomography (OPT) [5], [6], light sheet fluorescent microscopy [7]–[9], blockface imaging [10], [11], and serial two-photon tomography [4]. With many of these tools, cell types or gene products of critical interest can be visualized using transgenic optical markers, such as fluorescent proteins, under the control of appropriate promoters. New methods of optically clearing specimens will further expand the application of these techniques [12]. However, the appropriate transgenic mouse is not always available and it is impractical and expensive to generate such mice for studies where multiple markers are necessary simultaneously or where the breeding is already complicated due to the disease model being investigated.

Adaptation of staining methods with commercial antibodies, as used for traditional 2D immunohistochemistry, would provide much more flexibility to 3D optical imaging methods, enhance the impact and convenience of these tools, and enable routine analysis of cell and gene product distributions in 3D. Although antibody staining in 3D samples has been successful in some tissues [13], [14], it has posed challenges in the mouse brain due to low penetration of the antibodies, preventing the staining of cells deeper than a few hundred microns [15]. Therefore, we developed a straightforward antibody staining method that allows for penetration of antibodies in intact mouse brain samples. This method is flexible, can be used with a number of antibodies, allowing for the spatial distribution of multiple cell types to be assessed simultaneously, and is applicable to any 3D optical imaging modality. The staining method itself is simple and easy to apply, using a combination of heat, time, and specimen handling procedures available in most laboratories to increase antibody penetration into the mouse brain. Here we carefully evaluate the quality of the staining in mouse brain samples, focusing on neural progenitor cell distribution, and provide demonstrations of its potential and limitations for 3D visualizations.

## Materials and Methods

### Animals

All animal experiments were approved by the animal care committee for the Toronto Centre for Phenogenomics.

### Perfusion

Eight-week old male wildtype C57Bl6/J (Toronto Centre for Phenogenomics, in-house breeding, Toronto, Ontario, Canada) were anesthetized with an intraperitoneal injection of 150 mg/kg ketamine and 10 mg/kg xylazine.

#### 1% PFA perfusion

Anesthetized mice were perfused intracardially with 15 ml phosphate buffered saline (PBS, Wisent Bioproducts, Quebec, Canada) containing 10 U/ml heparin followed by 15 ml of 1% PFA. The brains were removed from the skull and soaked for 2 hours in 1% PFA and subsequently washed with PBS.

#### 4% PFA perfusion

Anesthetized mice were perfused intracardially with 30 ml PBS containing 10 U/ml heparin followed by 30 ml of 4% PFA. The brains were soaked in the skull overnight at 4°C. The brains were washed in PBS and removed from the skulls the following day.

### Diffusion of 150 kDa FITC-dextran

Samples approximately 4 mm in each dimension were cut using an adult mouse brain matrix (Kent Scientific Corp, Torrington, CT) and then incubated with 150 kDa FITC-dextran (Sigma, Ontario, Canada) for 5, 10, 24, or 48 hours at 4°C or 37°C. The samples were then sectioned into 50 µm sections on a vibratome (Leica, Germany), visualized using an inverted fluorescent microscope (Leica, Germany), and digitally captured using a cooled, CCD camera (Qimaging, BC, Canada). The microscope images were stitched together to visualize the entire section in a single image, and intensity curves representing diffusion of FITC-dextran into the sample were obtained by mapping the intensity along the line normal to the specimen edge and toward the centre of the section. Approximately 20 such linear intensities were obtained for each section. Subsequently, the linear distance along each curve was normalized by the square root of the incubation time. The intensity of each curve was also normalized, using a third-order polynomial fit to estimate the amplitude at the origin. After both the distance and intensity normalizations, equivalent curves were averaged together for visualization and plotted with 90% confidence intervals.

### 3D antibody staining of adult mouse brains

1% PFA perfused adult mouse brains were removed from the skull and then divided by removing the cerebellum and separating the hemispheres if desired. The samples were immediately dehydrated in a gradient of methanol solutions to 100% methanol over the course of one day. Immediate dehydration is important to preserve the cellular morphology of the lightly-fixed brain. The samples then underwent freeze/thaw (one hour at –80°C/one hour at room temperature) four times. The samples were rehydrated in a gradient of methanol solutions to PBS over the course of one day. The samples were mildly digested for 5 minutes using 10 mg/ml of Proteinase K (Promega, WI, USA) and then thoroughly washed to remove any residual Proteinase K. Antigen retrieval was performed using a Histos5 histology microwave (Milestone, MI, USA) in 0.01 M sodium citrate buffer pH 6 on an antigen retrieval program (0–90°C 11min, 90–98°C 3min, 98°C 10min). The samples were cooled to RT and subsequently washed in PBS to remove excess buffer. From this point, all incubations were performed in the Histos5 histology microwave. The samples were incubated at 37°C for 48 hours in primary antibody diluted in 5% normal goat serum (Jackson ImmunoResearch, PA, USA), 5% dimethylsulfoxide (DMSO, Fisher Scientific, Ontario, Canada), and 0.01% Triton X-100 (Bioshop Canada, Ontario, Canada). The samples were then washed at 37°C for 48 hours in 5% serum, 0.01% Triton X-100, replacing the solution once after 24 hours. Following this, the samples were incubated for 72 hours in secondary antibody diluted in 5% serum, 5% DMSO, and 0.01% Triton X-100. Finally, the samples were washed at 37°C for 48 hours in 0.01% Triton X-100, replacing the solution once after 24 hours.

Staining of single hemisphere brain samples using the described protocol for 3D imaging included the following: doublecortin (n = 4), GFAP (n = 4), nestin (n = 3), double stain of doublecortin and nestin (n = 1). Doublecortin was also used to stain a full mouse brain excluding the cerebellum (n = 2).

Control samples were generated using pre-absorbed primary antibodies when possible by incubating the primary antibody with a blocking peptide for 30 minutes at room temperature prior to staining. If no peptide for the primary antibody was available, the staining procedure was performed in the control except that the primary antibody was omitted during the appropriate step.

Staining was performed with the following antibodies: rabbit anti-doublecortin (Abcam), goat anti-doublecortin and peptide (Santa Cruz Biotechnology) rabbit anti-GFAP (Dako), chicken anti-nestin (Neuromics), goat anti-rabbit alexafluor 546 (Invitrogen), goat anti-rabbit alexafluor 488 (Invitrogen), goat anti-rabbit alexafluor 647 (Invitrogen), goat anti-chicken alexafluor 546 (Invitrogen), goat anti-chicken alexafluor 488 (Invitrogen), donkey anti-goat alexafluor 546 (Invitrogen), donkey anti-goat alexafluor 488 (Invitrogen).

### 2D immunostaining

Unstained or previously 3D-stained mouse brains were soaked in 30% sucrose to prepare them for cryosectioning. 10 µm cryosections were cut and the OCT was removed in a PBS wash. Antigen retrieval was performed at 90°C for 20 minutes followed by allowing the slides to cool to room temperature. The slides were washed 3x with PBS and once with 0.1% Triton X-100 and subsequently blocked with 5% normal goat serum. The slides were incubated with primary antibody (listed above) overnight at 4°C in 1% normal goat serum and 1% Triton X-100. The slides were then washed 5 minutes in PBS three times followed by a 1 hour incubation at room temperature in secondary antibody diluted in 1% normal goat serum, 0.1% Triton X-100. The slides were washed for 5 minutes in PBS three times followed a 5 minute wash in dH_2_O. Finally, the slides were rinsed with dH_2_O and mounted with Vectashield hard-set with DAPI mounting medium (Vector Labs, Ontario, Canada).

To test the primary and secondary staining steps of the 3D stain, slides from the previously stained brains were incubated with either both primary and secondary antibodies or secondary antibody only. All 3D staining used antibodies conjugated to alexafluor 546 and the follow-up restains used secondary antibodies conjugated to alexafluor 488.

### Sample preparation for imaging

#### Optical projection tomography (OPT)

Samples were embedded in a 1% low melting point agarose (Fisher Scientific, Ontario, Canada) in a cylindrical mold. The agar was allowed to solidify and the sample was dehydrated through a gradient of ethanol solutions. The sample was cleared in benzyl benzoate (Sigma, Ontario, Canada):benzyl alcohol (Fisher Scientific, Ontario, Canada) (2:1) over the course of 3 days. The cleared sample was imaged using OPT.

#### Serial two-photon tomography

After staining of samples fixed in 1%PFA as described above, samples were post-fixed overnight at 4°C in 4% PFA. This made the samples stiffer so that they could be cut more cleanly using the integrated vibratome of the serial two-photon tomography system. The following day, the samples were washed three times in PBS to remove any excess PFA and then stored overnight in PBS at 4°C. The samples were then embedded in 3% agarose (Bioshop Canada, Ontario, Canada) made with phosphate buffer [4].

### 3D Optical Imaging

#### OPT

Images were acquired on a custom-built OPT scanner [16], the details of which will be published separately. 1200 projection views were taken over 360°. Images were acquired using a λ = 425±15 nm excitation filter and a λ = 473 nm long-pass emission filter for autofluorescene imaging, λ = 531±20 nm excitation filter and λ = 593±20 nm emission filter for imaging alexafluor 546, and a λ = 628±20 nm excitation filter and λ = 692±20 nm emission filter for alexafluor 647 imaging with a 1024×1024 matrix on a cooled CCD camera (Qimaging, BC, Canada). The projections were reconstructed into the final 3D image using SkyScan NRECON Software (Bruker, Belgium).

#### Serial two-photon tomography

Images were acquired using a TissueCyte 1000 (TissueVision, Cambridge, MA, USA) serial two-photon tomography system using λ = 800 nm, 600 mW laser (Coherent, CA, USA) and a single-edge dichroic beamsplitter with λ = 552 nm with an in-plane resolution of 1.37 µm and a through-plane pixel resolution of 0.1 mm. Individual tiles were reconstructed in post-processing to generate the full 3D image.

### Visualization

A non-uniformity correction [17] of the autofluroescent OPT images was performed before display to correct for the apparently brighter intensity at the edge of these images that results due to light scatter and penetration. To approximate and remove the autofluorescence background from the stained image channel, a scale factor was computed by linear regression to match the autofluorescence channel (green) to the background of the stain (red). Subsequently, the scaled autoflurescence results were subtracted from the stain. A similar correction was also performed for the serial two-photon tomography images. For 3D display of some images, we applied a nonlinear low-pass filter to remove isolated high-intensity pixels (as described in the results for DCX and nestin staining). The isolated pixels were detected by performing a high-pass filter, normalizing the resulting image to the local standard deviation, and then thresholding to identify outliers. Subtraction of the high-pass filtered image from the original image where outliers were detected produced the filtered result for display and analysis.

### Density Maps

For GFAP serial two-photon tomography and histology images, cell area density was quantified and mapped in 2D slices for qualitative comparison. Images were blurred with a Gaussian filter and then cells were detected based on a high local intensity using both an intensity threshold and a local rank filter. Each identified pixel cluster was then represented with an average pixel location and taken to represent a single cell. An area density map was generated on a downsampled grid. At each grid point, the area density was computed by locating the eight nearest cell locations, and then using the average of their square distance to estimate the associated area.

## Results

### A simple protocol allows increased diffusion of macromolecules into the mouse brain

**Figure 1:**
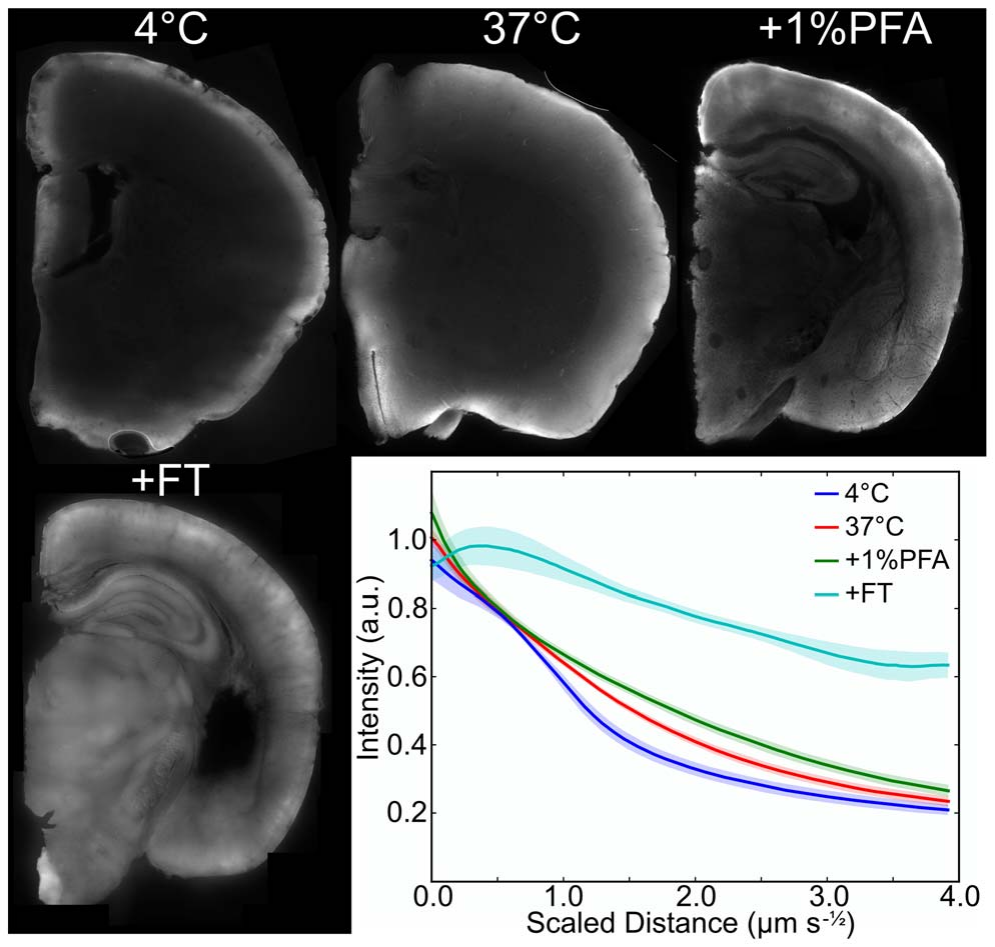
Dextran-FITC diffusion in brain specimens. 50 µm slices from a 4 mm hemisphere “stained” with dextran-FITC for 24 hours under different conditions as shown. Average one-dimensional intensity profiles from the edge of the tissue toward the center across several samples are shown at lower right with 90% confidence intervals. Samples processed for different lengths of time were combined by scaling the linear diffusion distance by the square root of time. The combination of increasing temperature, utilizing a 1% PFA fixation, and processing with a freeze/thaw, and proteinase K digestion (+FT) resulted in the biggest improvement to penetration.

We assayed diffusion of 150 kDa dextran FITC in ∼4 mm hemispheres of the mouse brain under various conditions in the aim of designing a protocol that would increase the penetration depth for antibody staining (Figure 1). After completing a “stain” with the dextran FITC, the brain specimen was cut on a vibratome and examined under a microscope. The standard protocol from which we started performed the “stain” at 4°C, in which diffusion appeared hindered, producing a band of concentrated fluorescence and a sharp boundary. We found that increasing the temperature to 37°C using a histology microwave increased the dextran FITC penetration and produced a fluorescence intensity profile more consistent with free diffusion. Decreasing the concentration of the fixative used from 4% paraformaldehyde (PFA) to 1% PFA modestly increased the depth of penetration further. Incorporating pre-staining processing steps that included freeze/thaw cycles and a short proteinase K digestion [15] increased penetration sufficiently to “stain” the half-brain sample in a reasonable time frame. We incorporated these steps into a 3D antibody staining protocol for the adult mouse brain that proceeded through the following steps: (1) 1%PFA fixation; (2) dehydration, freeze-thaw, rehydration and brief proteinase K digestion; (3) antigen retrieval (∼10mins at 98°C, ∼24mins total); (4) 48 hour soak in primary antibody (37°C); (5) 48 hour wash (37°C, change solution at 24 hours); (6) 72 hour soak in secondary antibody (37°C); and (6) 48 hour wash (37°C, change solution at 24 hours). When necessary, an optional final step can be added with an additional 4%PFA fixation to further stiffen tissue for cutting of thin sections for 2D or 3D imaging methods (i.e., variants of blockface imaging).

### “3D staining” in intact specimens produces similar results over much of the brain as traditional immunostaining

**Figure 2:**
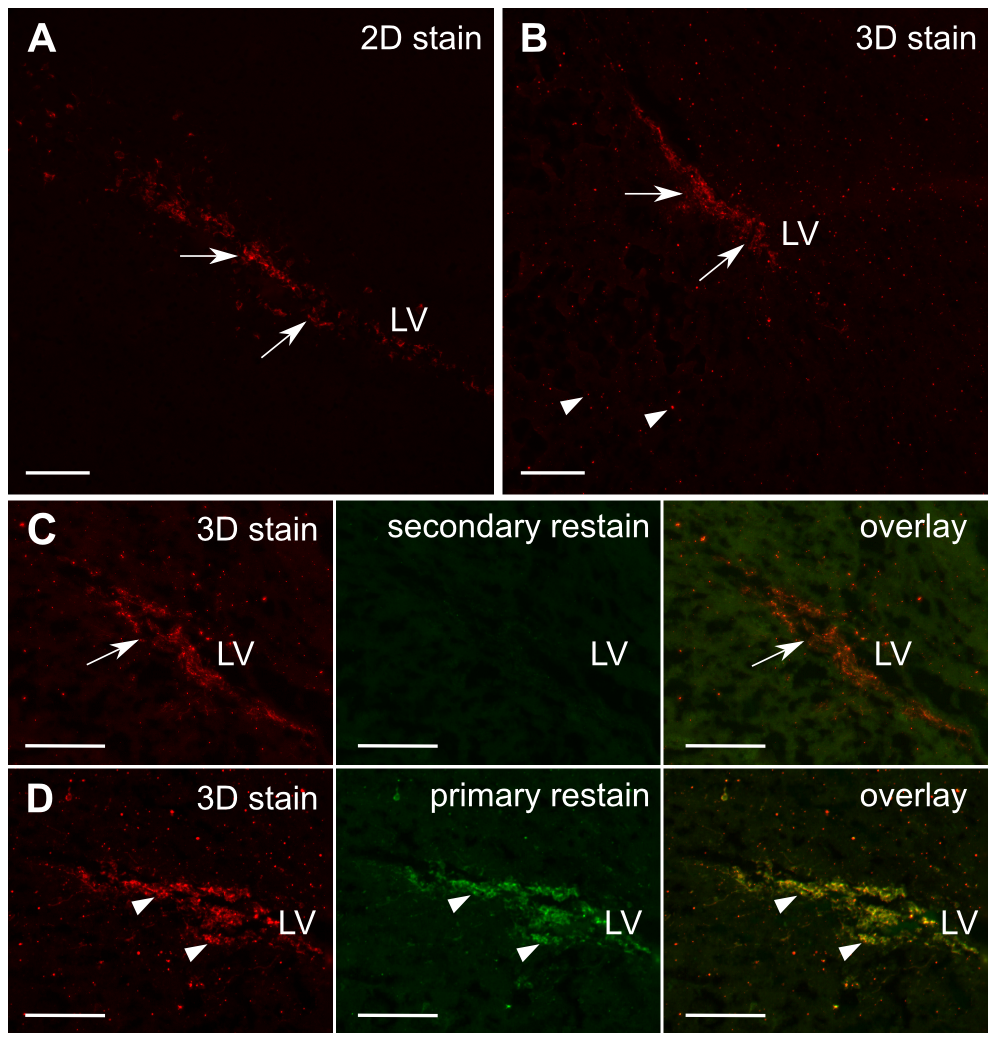
Validation of DCX staining. A traditional 2D stain of DCX (A) shows a reference distribution of migrating neuroblasts (red). The 3D staining method in a brain hemisphere (B) shows a comparable distribution (arrows, A and B), with the exception of some additional punctate fluorescence (arrowheads, B). Cryosections prepared from the 3D stained hemisphere were re-stained with another secondary antibody (green; C) to highlight primary antibody left unbound by secondary during the 3D stain; the primary antibody is bound by secondary in the 3D staining method (arrows). In (D), an additional cryosection from the same brain as (C) was re-stained with both primary and secondary antibody to highlight antigen binding sites not occupied by primary. Overlap of the 3D-stained and re-stained cells (arrowheads, D), indicate that no DCX cells have been missed in the 3D-stained images. LV = lateral ventricle, scalebars = 100 µm

**Figure 3:**
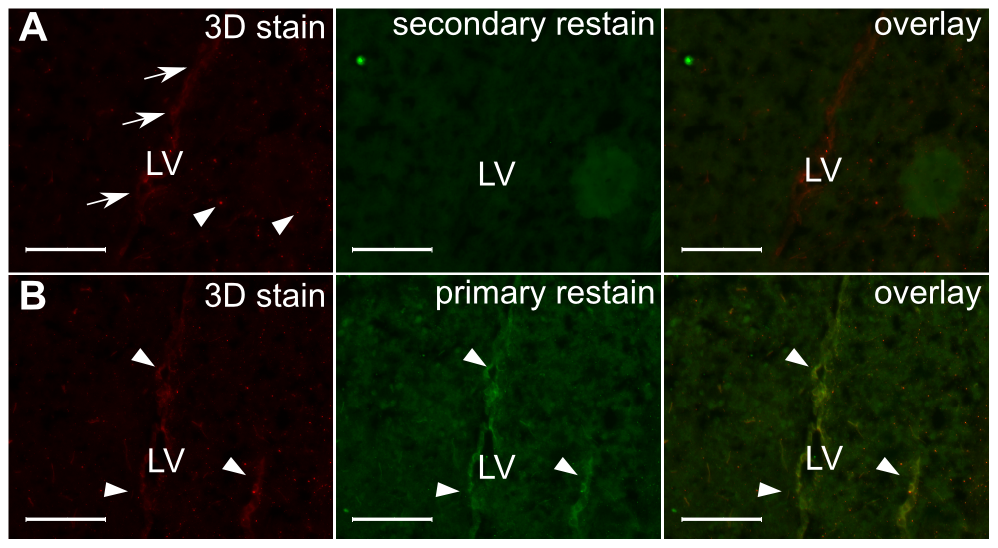
Validation of 3D nestin staining. Cryosections cut from a stained brain hemisphere show nestin-positive cells (red; arrows, A). Some additional punctate fluorescence was present (arrowheads, A). The sections were re-probed with a secondary antibody (green; A) to highlight primary antibody left unbound by secondary antibody. No evidence of primary antibody left unbound by secondary antibody was found. Sections were also re-stained with both primary and secondary antibodies (B) to highlight primary-antibody antigen sites unoccupied. There is overlap of the original 3D nestin stain with the 2D re-stain (arrowheads). LV = Lateral ventricle, scalebars = 100 µm.

**Figure 4:**
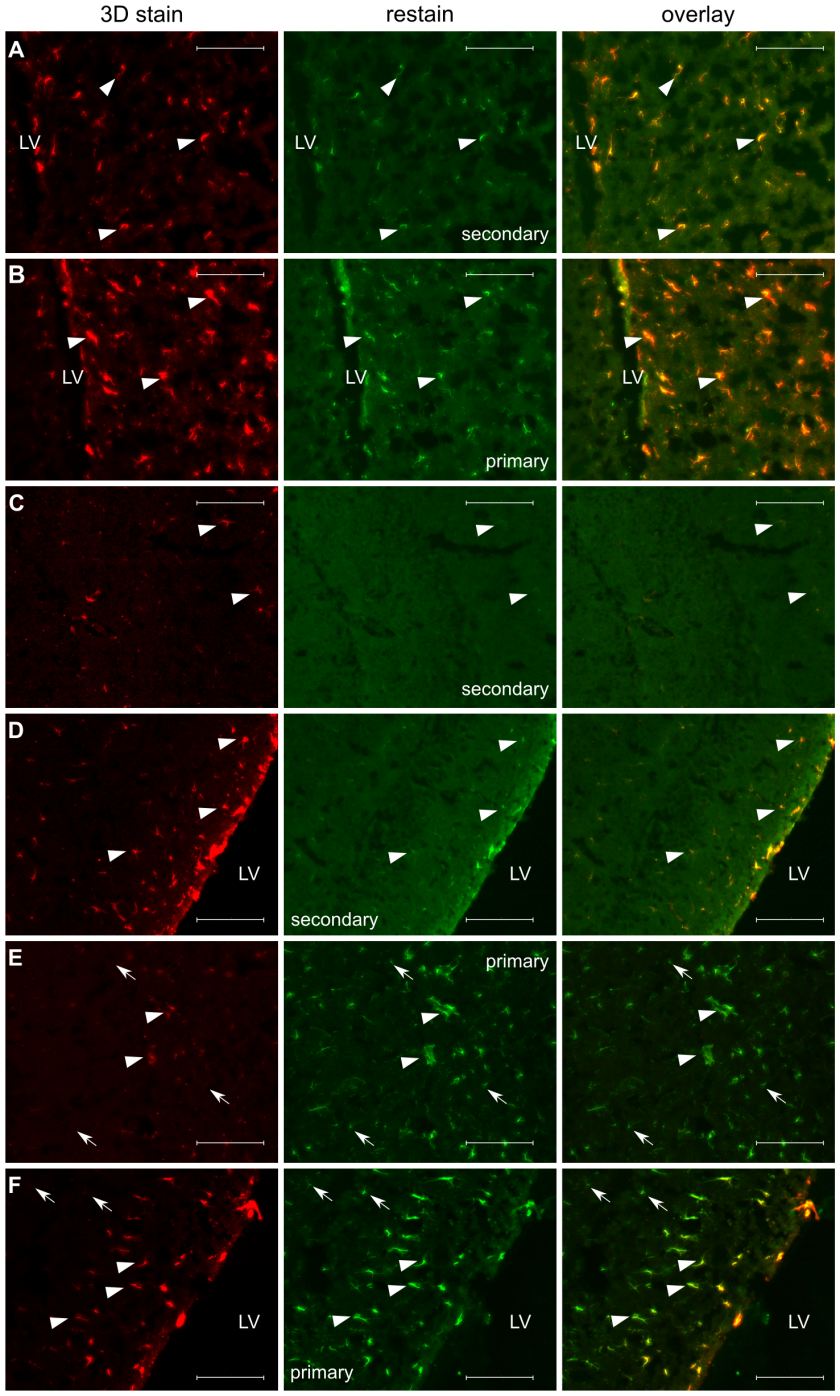
Validation of 3D GFAP staining. Cryosections from a the anterior (A, B) and posterior (C–F) of a GFAP-stained brain hemisphere are shown. Restain with another secondary antibody (green; A, C, D) highlights the presence of primary antibody left unbound by secondary antibody. Restain with both primary and secondary antibody (green; B, E, F) highlights the presence of additional antigen sites initially unoccupied by primary. Arrowheads highlight overlap of the re-stain and the original 3D stain. Arrows highlight cells that are only seen with the re-stain. In the anterior region, there remains complete overlap between the 3D stain (red) and the re-stains (A, B). In the posterior potion of the brain, there is overlap of the secondary antibody re-stain and the original 3D stain in the hippocampus (C) and posterior portion of the lateral ventricle (D). Re-stain with both primary and secondary antibody shows the presence of unoccupied primary antibody-antigen sites, including some cells not stained with the 3D method in the hippocampus (E) and by the posterior lateral ventricle (F). LV = lateral ventricle, scalebars = 100 µm.

We tested the 3D staining method with three different antibodies in single hemispheres from adult mouse brains and then sectioned the brains for comparison with traditionally-stained sections visualized with fluorescence microscopy. An example of these results is provided in Figure 2 for staining with doublecortin (DCX), which marks neuroblasts in the subgranular zone (SGZ) and the subventricular zone (SVZ), from which they migrate along the rostral migratory stream (RMS) to the olfactory bulbs [18]. We also evaluated 3D staining for nestin and glial fibrillary acidic protein (GFAP) (Figures 3 and 4 respectively). Nestin is a marker of neuronal stem and precursor cells in the adult mouse brain [19] and GFAP stains both astrocytes and neural stem cells [20].

Overall, we observed a similar distribution of DCX-staining in specimens prepared by the 3D staining method and then cryosectioned as we did in specimens prepared traditionally by first cryosectioning and then staining (Figure 2a and b). Likewise, 3D nestin staining showed the expected distribution of nestin-positive cells surrounding the lateral ventricles. 3D GFAP staining also showed comparable staining of white matter, such as the corpus callosum and mirrored the section obtained by traditional methods in the forebrain, although in more caudal regions staining was sparser and lighter than expected, particularly in the hippocampus. In the case of DCX and nestin, but not GFAP, we also observed high-intensity punctate fluorescence in the 3D stain not representative of staining in the traditional sections. The same punctate appearance can be replicated in a 2D-stained section by mixing primary and secondary antibody in solution prior to “staining”, suggesting that the punctate voxels we observed are likely due to unreacted primary after an incomplete wash step. Lengthening the wash step or decreasing the primary antibody concentration could reduce these artifacts, though at the cost of increased sample preparation times or incomplete staining. As an alternative, to keep the sample preparation time short in 3D imaging applications, these pixels/voxels can also be identified as isolated high-intensity pixels/voxels and then be filtered from the image on this basis.

We subsequently examined the 3D staining in more detail by re-staining sections with traditional methods after first preparing them with the 3D method in intact brain hemispheres. First, subsets of sections were re-stained with a secondary antibody conjugated to a different color fluorophore to visualize regions where primary antibody had not been properly visualized by secondary antibody. Red secondary antibodies were used for the 3D stain, and green secondary antibodies were used for the re-stain in individual sections. We observed that in the cases of DCX and nestin, there was no primary antibody that remained unbound by secondary antibody, so that little or no green secondary could be observed (Figure 2C, Figure 3A). On the other hand, a re-stain with green secondary antibody for GFAP did show regions where additional binding took place. In the forebrain, there was complete overlap in the green staining and the red 3D stain (Figure 3A), indicating that penetration of the secondary antibody was adequate to show the complete cell distribution but that additional secondary antibody binding sites were available. In more caudal regions of the brain, there were some cells where the 3D staining labeled GFAP-positive cells, but were only faintly visualized. The re-stain in sections with green secondary antibody only showed modest binding, suggesting that penetration of secondary antibodies during the 3D stain was not limiting visualization of thse cells (Figure 4C and D).

Finally, another subset of sections cut from specimens stained with the 3D method were re-stained with both primary and secondary antibody to visualize any regions left unstained by the primary during the 3D staining procedure. In the case of DCX and nestin, restained regions were observed, but found to overlap with the 3D stain (Figure 2D, Figure 4B), indicating that cells had been adequately labeled by primary but that some additional antigen sites were left unoccupied by the original primary antibody. For GFAP, the anterior region of the brain similarly showed no unstained cells (Figure 4B). However, in more caudal brain regions, the re-stain in sections with both primary and green secondary showed that there were some areas where the primary antibody penetration was incomplete, specifically in the hippocampus where some cells showed only green fluorescence from the re-stain (Figure 2E and F). This indicates that, using the unmodified protocol we assayed, the GFAP primary antibody was slower to penetrate the posterior region of the brain and this resulted in poorer staining in parts of the hippocampus.

### Antibody staining can be visualized in brain samples with 3D optical imaging

**Figure 5:**
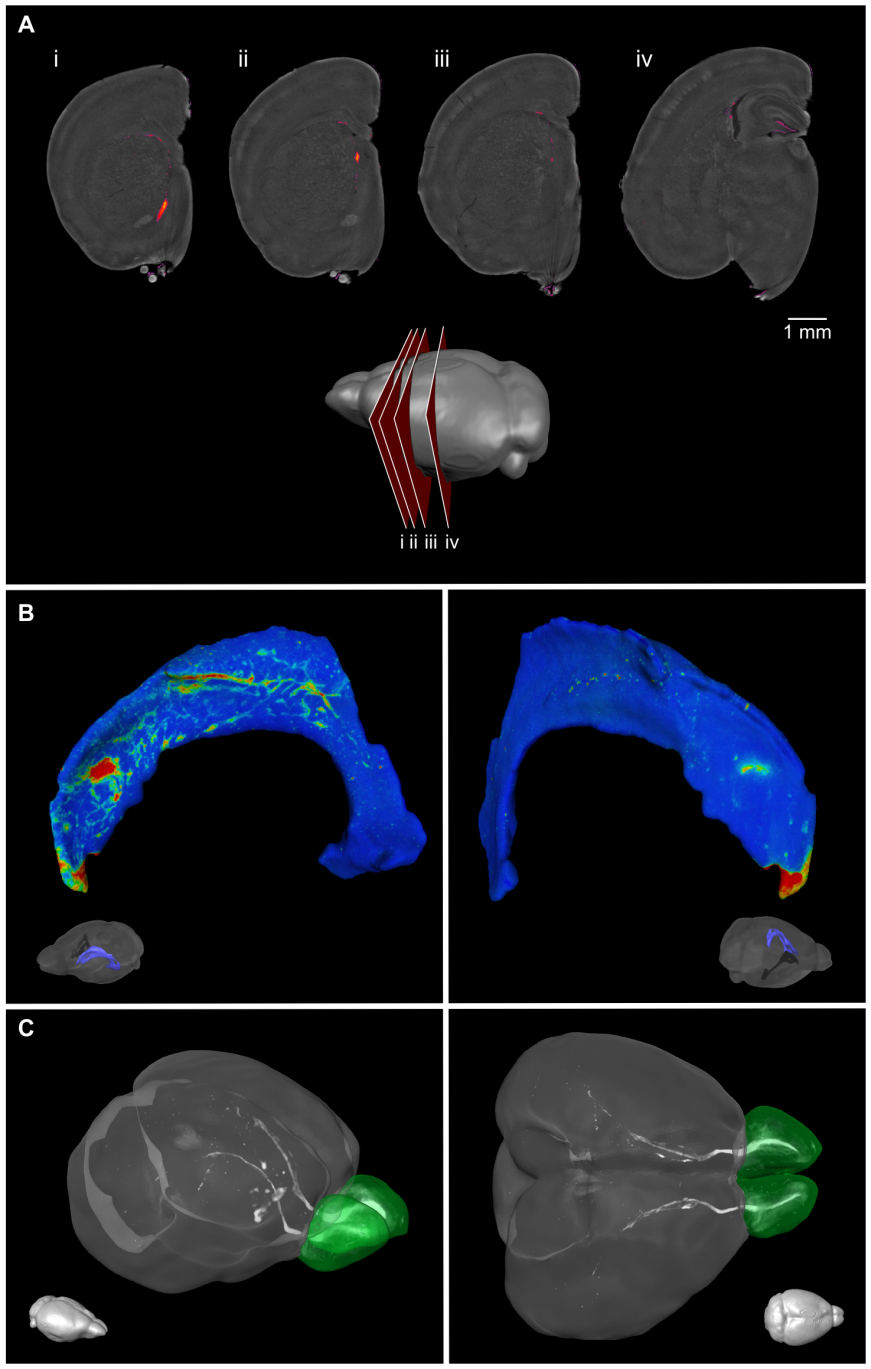
3D OPT imaging of DCX staining in the mouse brain. 3D OPT of a DCX-stained single hemisphere are shown as optical slices. DCX staining is visible surrounding the lateral ventricle (A; i–iii) and in the subgranular zone of the dentate gyrus (A; iv). A cartoon shows the location of the slices. The chains of DCX migrating neuroblasts along the lateral ventricle can be visualized on the ventricle surface in 3D (B). In (C), a maximum intensity projection of a full brain shows cells migrating along the RMS to the olfactory bulbs (shaded green).

**Figure 6:**
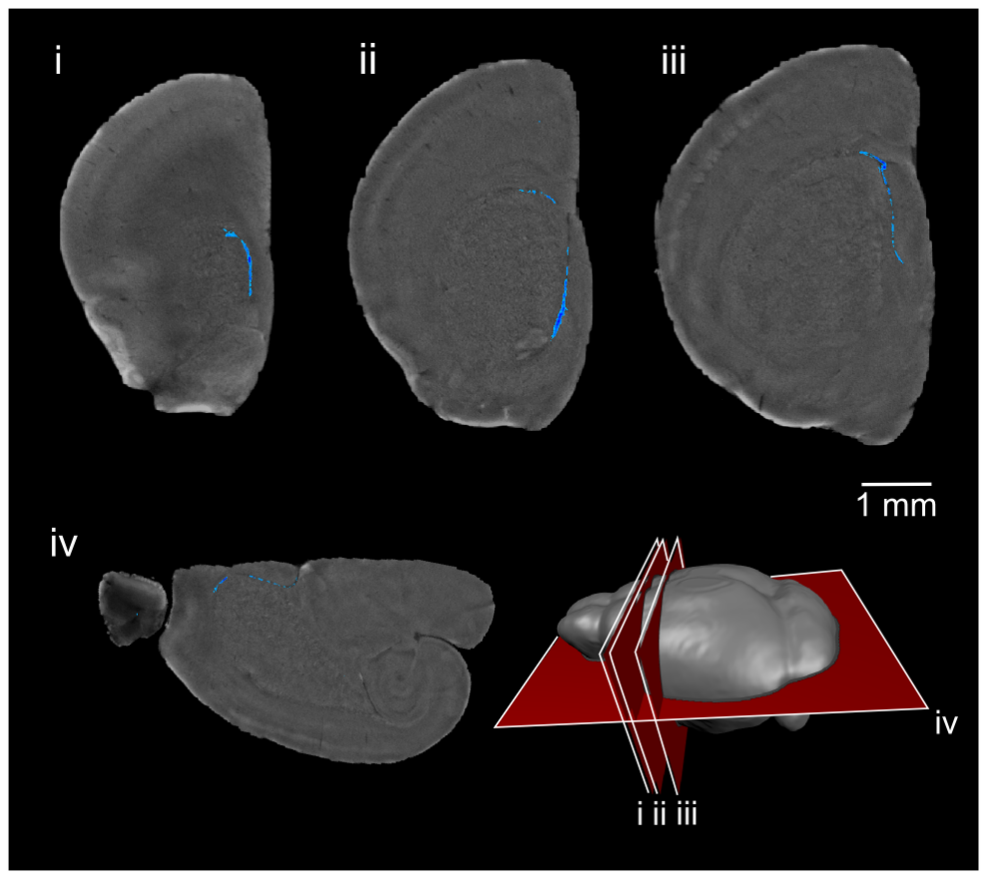
3D OPT imaging of nestin staining in the mouse brain. 3D optical imaging with OPT was used to image a nestin-stained brain hemisphere. Optical slices of the brain are shown and nestin-positive cells are visible surrounding the lateral ventricle. A cartoon shows the location of the slices.

**Figure 7:**
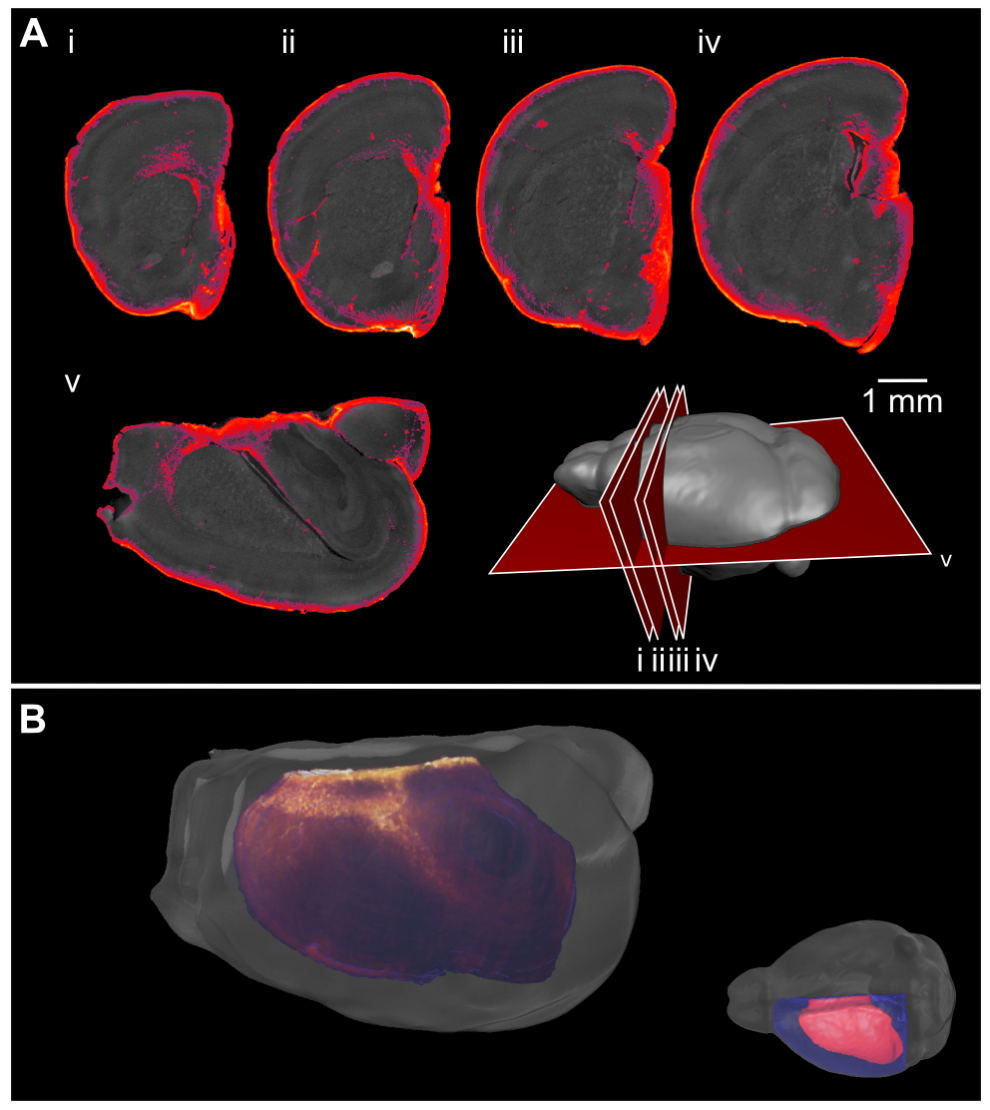
3D OPT imaging of GFAP staining in the mouse brain. 3D optical imaging with OPT was used to image a GFAP-stained brain hemisphere. Optical slices of the brain are shown and GFAP-positive cells are seen throughout the brain (A). A cartoon shows the location of the slices. A 3D representation shows the distribution of GFAP staining along the corpus callosum (B).

The key benefit of doing 3D staining is the ability to visualize the distribution of the stained cells in a larger anatomical context. This allows appreciation of cell distributions that are difficult to assess in 2D, such as, for example, the chains of DCX-positive migrating neuroblasts. In Figure 5, optical slices from an OPT image of a single hemisphere stained with DCX are shown with autofluorescence in grayscale to show anatomy and a color overlay showing DCX stain. DCX-positive cells are found in the expected areas of the brain including the RMS, SVZ surrounding the lateral ventricles, and the SGZ in the hippocampus (Figure 5A). Projection of the DCX intensities onto a surface rendering of the lateral ventricles shows the migrating chains of DCX-positive cells on the lateral face (Figure 5B), consistent with expectations from published work [18]. A maximum intensity projection of DCX staining in a whole mouse brain (without the cerebellum) shows the distribution of the stain in full anatomical context and allows the migrating chains from the SVZ of the lateral ventricles along the RMS to the olfactory bulbs to be visualized (Figure 5C). Similarly, optical slices of nestin- and GFAP-stained brains imaged with OPT demonstrate the application of this method for other antibodies (Figures 6 and 7). As is common practice for traditional antibody staining in thin sections, a 3D control stain was also performed for each stain to verify specificity by staining with either a pre-absorbed primary antibody or a secondary antibody in the absence of primary antibody.

**Figure 8:**
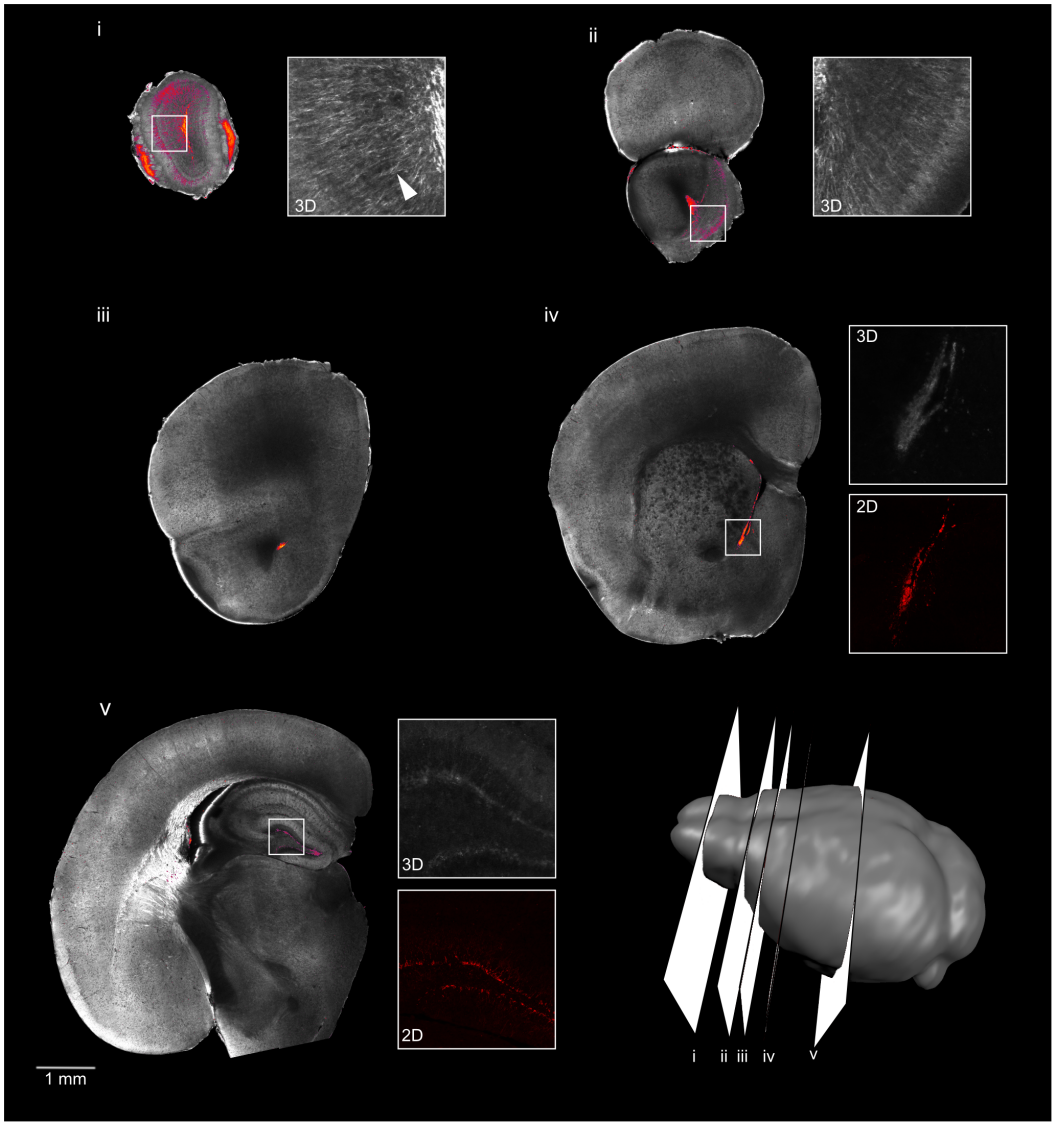
3D serial two-photon tomography imaging of DCX staining in the mouse brain. Serial two-photon tomography was used to image a single hemisphere stained with DCX. Five individual slices are shown, with the slice location shown in the cartoon at lower right. For each slice, the grayscale image shows autofluorescence for anatomical reference and the color overlay shows the DCX staining. Neuronal processes can be distinguished (DCX in grayscale insets i, ii; arrowhead, inset i). The staining pattern is comparable to traditional 2D sections (insets with red color, iv and v), although with lower contrast to background signal.

**Figure 9:**
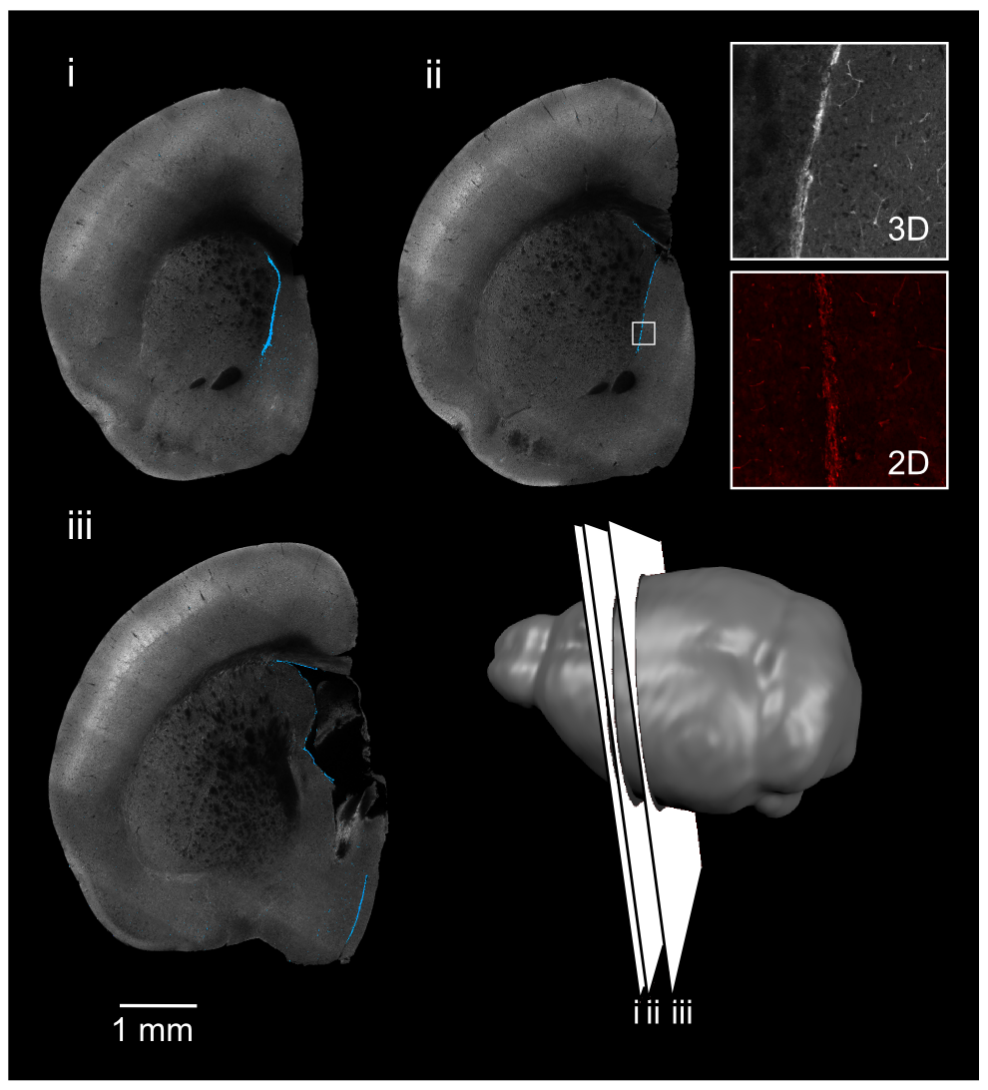
3D serial two-photon tomography imaging of nestin staining in the mouse brain. Serial two-photon tomography was used to image a single brain hemisphere stained with nestin. The slice locations are shown in the cartoon. The staining pattern (grayscale inset) is comparable to the 2D histology section (color inset).

**Figure 10:**
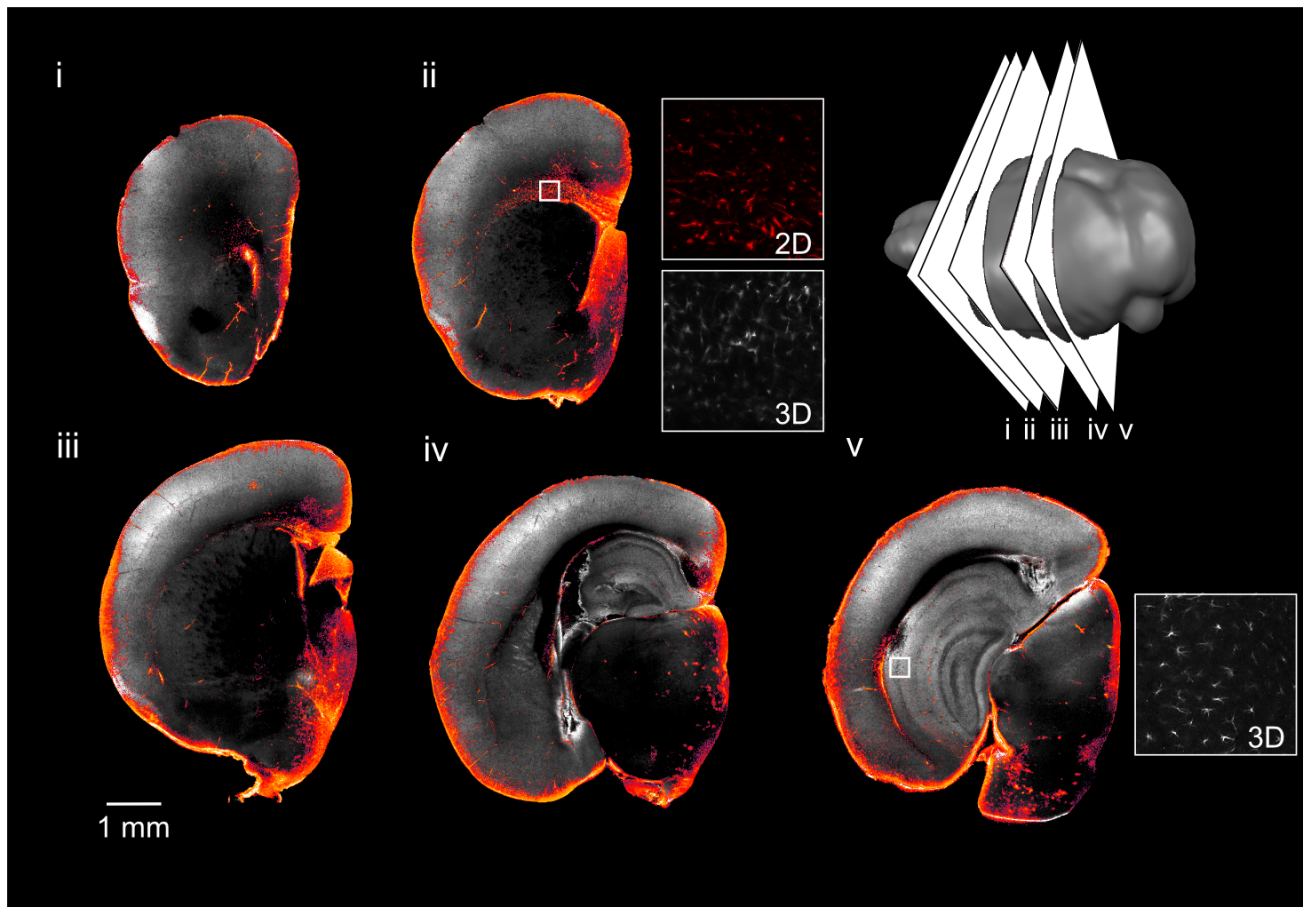
3D serial two-photon tomography imaging of GFAP staining in the mouse brain. Serial two-photon tomography was used to image a single hemisphere stained with GFAP. Optical slices through the brain show the staining pattern, which is widespread throughout the brain. The slice location is shown in the cartoon. The grayscale inset shows the typical astrocyte morphology, which is comparable to the 2D histology section (colour inset).

**Figure 11:**
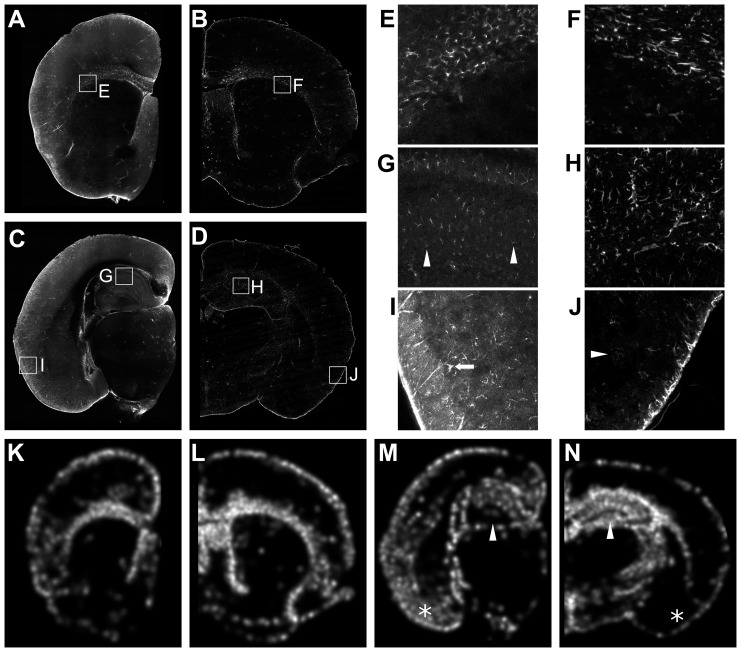
Comparison of serial two-photon tomography in GFAP-stained brains and traditional immunohistochemistry in sections. Slices from a 3D serial two-photon tomography data set (A, C) are compared with traditional sections (B, D). Expanded images are shown for comparable regions (E, G, I and F, H, J). Rostral sections (A, B and E, F) show comparable results. Staining in the hippocampus is lighter in the 3D stained section (G) than in the traditional section (H). At the periphery, the 3D stain produces more intense GFAP signal with a higher background (I) than does the traditional section (J). Examples of lightly-stained cells are highlighted with arrowheads in (G) and (J). The arrow in (I) highlights an example of the characteristic astrocyte morphology. Maps of cell density are shown in (K, M). Rostral cell densities (K, L) are similar between the two methods. In the caudal regions (M, N), the density in the dentate gyrus (white arrowheads) is reduced in the 3D stain while it is appears increased in some regions of the cortex (asterisks). Cell density maps (K–N) are shown with black and white corresponding to <30 cells/mm^2^ and >1200 cells/mm^2^ respectively.

The staining method as described above for OPT can be readily applied to other 3D optical imaging modalities. To demonstrate this, we imaged DCX-, nestin- and GFAP-stained samples using serial two-photon tomography [4], a technique that combines block face imaging with two-photon microscopy and increases the pixel resolution to ∼1 µm. This resolution is comparable to 2D histological sections. The distributions of DCX-, nestin-, and GFAP-positive cells are found in the expected areas and mirror the OPT results (Figures 8, 9, and 10 respectively). However, the additional resolution obtained with two-photon tomography allows one to appreciate the cellular morphology as well, including neuronal processes (Figure 8) and astrocyte morphology (Figures 10 and 11).

The additional cellular resolution of serial two-photon tomography (as compared to OPT) can be exploited for quantification, such as cell counting in an extended volume. As evaluation of the 3D antibody staining in sections indicated that the penetration of the GFAP antibody was not as complete as the DCX and nestin antibody penetration, we compared the quantification of cell density in the two cases. We generated 2D area density maps from individual slices of the serial two-photon tomography GFAP volume images and from individual GFAP-stained histological sections (Figure 11). A zoomed in image of the corpus callosum shows similar staining for the 3D immunostaining method in comparison to the 2D method (Figure 11E and F); however, there is fainter and less abundant staining in the hippocampus and dentate gyrus for the 3D method in comparison to traditional immunostaining (Figure 11G and H), matching the previous staining results. The density maps produced from the 3D staining method can be compared to those obtained with traditional histology (Figure 11K and 11M vs 11L and 11N). In the anterior portion of the brain, the density maps are very similar. In the posterior portion, the density maps show that the 3D staining, as applied here, is not detecting as many cells in parts of the hippocampus (particularly in the dentate gyrus), but shows an increased staining in the cortex in comparison to the 2D stained tissue (Figure 11M,N).

### 3D double staining enables visualization of two different cell types simultaneously in the adult mouse brain

While the ability to visualize the distribution of a single cell population in a 3D context will be useful in its own right in many studies, it is advantageous to be able to investigate the spatial distribution of two cell types in relation to one another. For this reason, we used the 3D staining method for simultaneous staining with two antibodies, a common practice with traditional immunohistochemistry.

**Figure 12:**
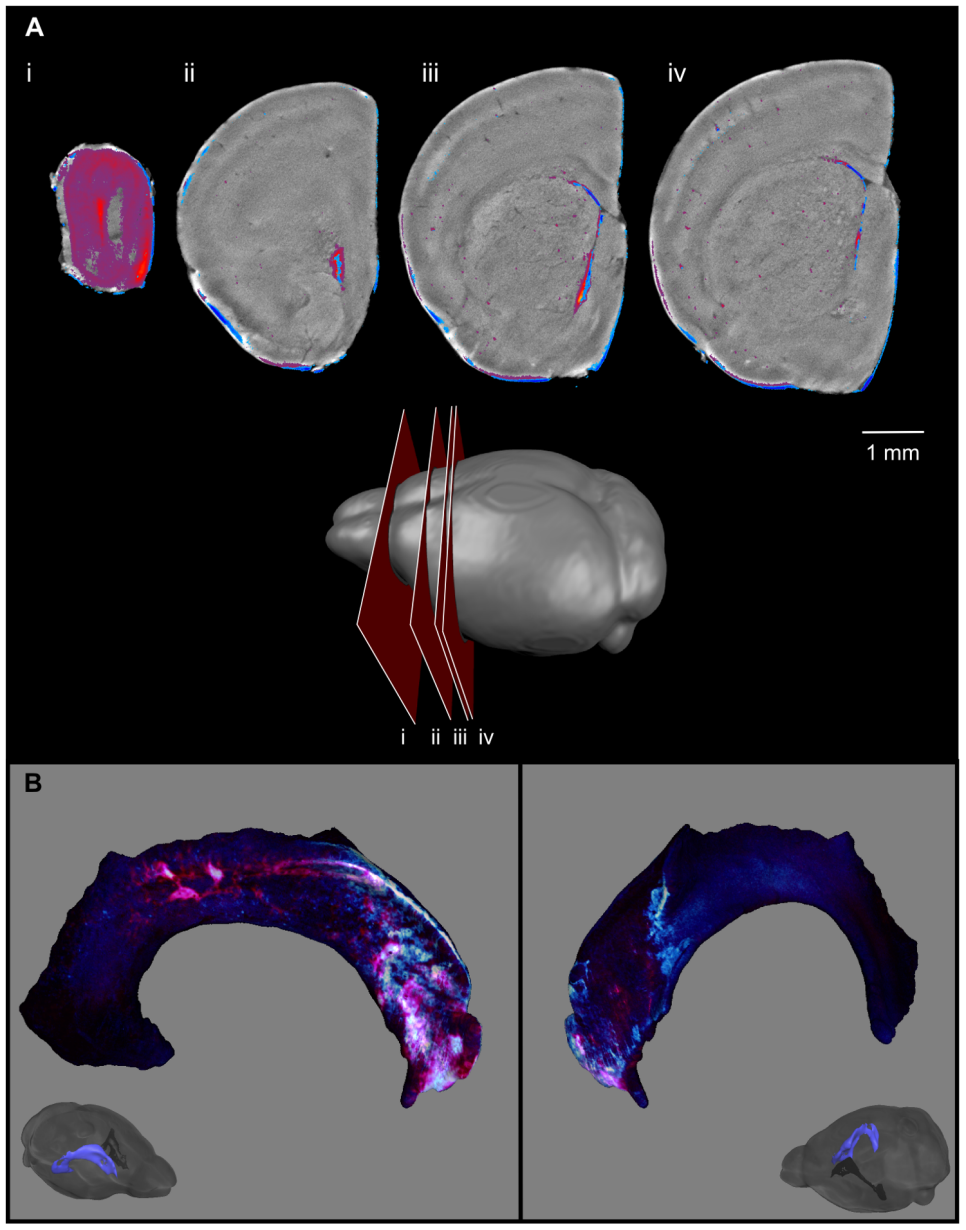
3D OPT imaging of DCX and nestin stained mouse brain. 3D optical imaging with OPT was used to image a single hemisphere stained with DCX (red) and nestin (blue) simultaneously. Optical slices through the tissue are shown to represent the 3D nature of the sample (A). The slice orientation is shown on a cartoon. A 3D representation shows the distribution of DCX- and nestin-stained cells along the surface of the lateral ventricle (B).

We used the nestin and DCX antibodies demonstrated previously and expanded the staining protocol by running the staining procedure twice in series (i.e., adding a second primary antibody soak, wash, secondary antibody soak, and wash following the original protocol with the first antibody, using the same times and temperature). We then imaged the specimens with OPT to visualize the resulting stain for each cell population and the autofluorescence background to provide anatomical context (Figure 12).

## Discussion

The vast majority of mouse studies rely on histology to assess cellular events in disease, development, or aging. In particular, the ability to visualize neural progenitor and related cells in 3D allows for their spatial distribution to be analyzed over a large volume and the results to be placed in a larger anatomical context. We have established a staining procedure that allows for the mapping of doublecortin-, nestin-, and GFAP-positive cells in the SVZ, SGZ, RMS, olfactory bulbs and cortex.

The protocol that we developed shares some similarities with other reported protocols. In particular, the freeze/thaw cycle and mild digestion of the extracellular matrix have been reported elsewhere [13], [15], [21]. Our protocol additionally used a light fixation (1% PFA rather than 4% PFA) and performed incubations at 37°C, rather than at either 4°C or room temperature. Both these steps provided improved diffusion of antibodies into the tissue. Note that, while we performed these incubations in a histology microwave, it may be possible to the carry out the protocol in a temperature-controlled waterbath as well. This means that the 3D staining protocol can be implemented without any specialized equipment or reagents and that it uses only commercially available antibodies.

Generally speaking, the protocol we applied generically to all three of the antibodies described should be adapted for each new antibody. This empirical adjustment is already common practice in traditional immunohistochemistry. The selection of the antibody dilution and the duration of each step will likely depend on the antibody, the abundance of the protein, and the location of structures of interest. For example, we found that the concentration of DCX antibody needed to be higher to visualize the SGZ and the olfactory bulb then it did if only the SVZ and RMS were of interest. Similarly, GFAP penetration to posterior brain regions was slow, which we attribute largely to the high concentration of tissue antigen for GFAP, particularly in the pial layer surrounding the brain [22]. The concentration of antigen likely also explains the difference in the appearance of some punctate voxels/pixels between the GFAP and DCX/nestin images. Thus, the region of interest and the concentration of antigen may be an important consideration in selecting antibodies for 3D staining and in adapting the proposed 3D staining method. Once the final concentrations and antibodies have been selected, we have found the protocol to be very repeatable for subsequent specimens.

The combination of immunostaining with 3D optical image acquisition provides new opportunities for quantification of cell distributions across populations. Specifically, the acquisition of 3D digital volumes enables population comparisons through use of computer automated registration for alignment of multiple image data sets [1], [23], [24]. Anatomical features (obtained, for instance, from autofluorescence) can provide assessment of morphological phenotypes, and then the antibody specific image can provide quantification of underlying changes in cell populations. Development of such an automated and comprehensive quantification will permit studies that might otherwise be prohibitively laborious.

Clearing of samples is a critical component of imaging with OPT, LSFM or related techniques. In this work, stained brains were cleared with BABB for imaging with OPT. Other processes and agents have been reported that are better able to preserve the fluorescence from transgenically-expressed proteins and may pose some advantages for antibody staining as well [12], [25], [26]. A new method called CLARITY, in particular, was published by the Deisseroth group while this manuscript was in preparation. The authors demonstrate immunostaining in the clarified brain. The diffusion of antibodies into the brain was undoubtedly enhanced by the removal of lipids in the CLARITY method. The simple modifications for staining that we have presented here are complimentary; the extended incubation times for immunostaining even with CLARITY can be further reduced by appropriate selection of the concentration of formaldehyde/hydrogel and by adjustment of the incubation temperature as described here. Similarly, the processing associated with CLARITY may improve the diffusion that we have observed, which was not complete in some cases. Short and reliable immunostaining methods for intact brains will extend the applicability of 3D optical imaging in future studies.

Although we demonstrated brain imaging with OPT [1], [5] and serial two-photon tomography [4], we anticipate that this method could readily be applied to other 3D optical imaging modalities (such as LSFM [7]–[9] or other block-face imaging techniques [10], [11]). There are, of course, advantages and disadvantages to each imaging type. In our case, the OPT imaging procedure itself is very fast and the sample can be recovered for further analysis if desired. On the other had, serial two-photon tomography is able to achieve higher resolution, and is much less affected by the autofluorescence background or by depth within the tissue (owing to serial cutting). In either case, the 3D staining method is readily applicable in any laboratory with access to appropriate 3D optical imaging tools and will expand the potential impact of these tools in biomedical research.
